# Diurnal and daily fluctuations in levels of the urinary oxidative stress marker 8-hydroxyguanosine in spot urine samples

**DOI:** 10.1186/s41021-025-00324-0

**Published:** 2025-01-22

**Authors:** Yun-Shan Li, Koichi Fujisawa, Kazuaki Kawai

**Affiliations:** 1 Department of Environmental Oncology, Institute of Industrial Ecological Sciences, University of Occupational and Environmental Health, Japan, 1-1 Iseigaoka, Yahatanishi-ku, Kitakyushu, 807-8555 Japan; 2 Center for Stress-Related Disease Control and Prevention, University of Occupational and Environmental Health, Japan, 1-1 Iseigaoka, Yahatanishi-ku, Kitakyushu, 807-8555 Japan

**Keywords:** Daily fluctuation, Diurnal variation, Oxidative stress, Urine, 8-Hydroxyguanosine

## Abstract

**Background:**

Urinary 8-hydroxyguanosine (8-OHGuo) levels serve as a biomarker for oxidative stress and hydroxyl radical–induced RNA damage. Evaluating the diurnal and daily fluctuations in urinary 8-OHGuo excretion levels is essential for understanding its implications. However, research in this area remains limited. In this study, we aim to investigate the diurnal and daily fluctuations in 8-OHGuo levels as well as the factors that influence these variations, using spot urine samples.

**Methods:**

Urine samples were collected from seven healthy participants during each urination from the time of awakening until 24:00 h to evaluate diurnal variations. To assess daily fluctuations, urine samples were collected from 18 healthy participants at the time of awakening for 23 consecutive days. The urinary 8-OHGuo levels were measured using an HPLC-ECD method.

**Results:**

No significant variations were observed in the diurnal levels of urinary 8-OHGuo among non-smokers. Conversely, the daily variation of 8-OHGuo in the urine of the smoker was significant, with a coefficient of variation of 18.71%. Each individual maintained a characteristic value despite some diurnal fluctuations. Furthermore, the daily levels of 8-OHGuo exhibited a range of variations influenced by lifestyle factors, including mental state, sleep duration, smoking, menstrual cycle, and dietary habits.

**Conclusion:**

As a specific marker of RNA oxidation, 8-OHGuo provides unique insights distinct from those provided by the widely used DNA oxidation marker 8-hydroxydeoxyguanosine as an indicator of oxidative stress. Urinary 8-OHGuo could serve as a valuable biomarker for managing and preventing oxidative stress–related diseases, provided that the specific range of daily variations is established. The high daily variation in urinary 8-OHGuo levels necessitates the use of multiple samples to accurately determine individual levels. However, further research with large sample sizes will help to validate these findings.

## Introduction

Oxidative stress refers to an imbalance between reactive oxygen species (ROS) and the biological antioxidant system, in which excess ROS damage cells and tissues, adversely affecting nucleic acids, lipids, and proteins essential for normal cellular function. Oxidative stress can induce or exacerbate various diseases, including lifestyle-related conditions and cancer [1]. ROS are generated because of various environmental factors, occupational exposures, mental stress, and cellular oxygen metabolism [2–4]. ROS are considered essential for cell functions and the regulation of signal transduction pathways [5, 6]. However, ROS are short-lived and challenging to measure owing to their chemical nature as radical compounds with an unpaired electron. Consequently, analyses typically focus on sufficiently stable end products from the oxidation of damaged biomolecules, such as lipids, proteins, and nucleic acids, which serve as biomarkers [6]. 8-Hydroxyguanosine (8-OHGuo), a ribonucleoside formed as an RNA adduct by ROS in tissues and urine, is regarded as a reliable marker for assessing oxidative stress [7–9]. The synthesis of primary protein structure relies on genetic information encoded in mRNA; however, the oxidation of mRNA diminishes the effectiveness of translation and can lead to the production of abnormal proteins, contributing to cell death [10]. Broedbaek et al. [11] reported a significant positive correlation between urinary levels of 8-hydroxy-2'-deoxyguanosine (8-OHdG), a widely used biomarker of oxidative DNA damage, and 8-OHGuo. Furthermore, both 8-OHGuo and 8-OHdG levels were found to be higher in smokers than in non-smokers [12]. Notably, a significant increase in 8-OHGuo was observed in liver RNA following doxorubicin administration in rats, whereas no corresponding increase in 8-OHdG in DNA was noted [13]. The urinary 8-OHGuo levels in patients with osteoporosis and nephrotic syndrome are significantly higher than those in normal individuals, whereas no difference is observed in 8-OHdG levels [14, 15]. Yamauchi et al. found that the 8-OHdG level in the urine of patients with acute arsenic poisoning did not significantly increase during the first week of exposure to arsenic trioxide but reached its peak value after 30 days of exposure [16]. Some studies suggest that the level of 8-OHGuo, associated with oxidized RNA, increases in the earliest stages of certain diseases and shortly after occupational exposure [17, 18]. One possible reason is that single-stranded RNA has a more open structure than double-stranded DNA and lacks the protective features of double helices and histones, making it more susceptible to oxidative damage. This suggests that 8-OHGuo may be a more effective indicator of oxidative stress than the commonly used DNA oxidative damage marker, 8-OHdG, in the earliest stages of certain diseases and shortly after occupational exposure. When analyzing the urinary levels of 8-hydroxyguanine (8-OHGua), a commonly used biomarker for evaluating oxidative stress, the dietary intake of 8-OHGua must be considered, as studies show that 90% of dietary 8-OHGua administered to rats is excreted in urine [19, 20]. Therefore, when conducting research involving humans, it is essential to minimize the influence of dietary 8-OHGua, particularly from fish products rich in 8-OHGua, prior to urine collection. However, the level of neither 8-OHGuo nor 8-OHdG is affected, or is only minimally affected, by diet [19–21]. Therefore, urinary 8-OHGuo is commonly measured to assess the extent of oxidative RNA damage due to the noninvasive nature of collection and the stability of the sample, which minimizes the risk of artefactual production. Additionally, urinary 8-OHGuo is considered a good indicator of whole-body oxidative stress because it may be produced from the hydrolysis of 8-OHGTP by the sanitizing enzyme, MTH1, contributing to its excretion, or from the degradation of oxidized RNA into urine [22]. The levels of 8-OHGuo in spot urine samples have been utilized as biomarkers of oxidative stress to assess the adverse health effects of occupational and environmental exposure [18, 23, 24]. Andreoli et al. [25] proposed that normalizing biomarkers to creatinine levels in spot urine samples could help mitigate significant intra- and inter-individual variations in diuresis, as well as differences in lean body mass and physical activity levels. Consequently, creatinine levels are commonly used for normalization when assessing indicators of oxidative stress in spot urine samples. However, a debate still exists regarding whether a single spot urine sample is sufficient to accurately reflect an individual’s oxidative stress level. Assessing the biological and methodological variability of urinary 8-OHGuo excretion is a key aspect in establishing its utility as a clinical biomarker. Particularly, it is important to evaluate the diurnal and daily fluctuations in urinary 8-OHGuo excretion. It would be highly beneficial if 8-OHGuo levels in spot urine could be used as reliable indicators of oxidative stress. However, research in these areas remains limited. To reassess the usefulness of spot urine 8-OHGuo levels for oxidative stress estimation, we aimed to investigate the diurnal and daily fluctuations as well as factors influencing 8-OHGuo in spot urine samples.

## Materials and methods

### Chemicals and reagents

8-OHGuo (> 98%) was purchased from Abcam PLC (Cambridge, UK). Creatinine (99.0%) was purchased from Wako Pure Chemical Industries (Osaka, Japan). HPLC-grade methanol and acetonitrile were procured from Wako Pure Chemical Industries and Kanto Chemical Co. (Tokyo, Japan), respectively.

### Collection of urine samples

To assess the diurnal variations in urinary 8-OHGuo levels, urine samples were collected from seven healthy participants (five non-smokers: four men and one woman, aged 21 to 51 years; one smoker: man, aged 21 years; and one ex-smoker: man, aged 22 years) during each urination from the time of awakening until midnight (24:00). To evaluate daily variations in urinary 8-OHGuo, urine samples were collected from 18 healthy participants (11 men and 7 women, aged 21 to 51 years) for 23 consecutive days at the time of awakening. Throughout the study, the participants’ lifestyle factors, including diet, sleep patterns, smoking, and other habits, were monitored. The diet and lifestyle of the participants were not regulated. Urine samples were stored at − 20 °C until analysis. Written informed consent for publication was obtained from all participants, and the study protocol was approved by the Ethics Committee of Medicine and Medical Care of the University of Occupational and Environmental Health, Japan (Approval No. R3-016).

### Measurement of urinary 8-OHGuo

Urinary 8-OHGuo levels were measured according to a previously described method, with slight modifications [26]. Briefly, urine samples were thawed to 25 °C and centrifuged at 3,500 × g for 10 min at 25 °C. A 50-μL aliquot of the urine supernatant was mixed with 20 μL dilution solution containing 4% acetonitrile in a solution of 130 mM NaOAc (pH 4.5) and 0.6 mM H_2_SO_4_. Then, a pre-treatment filter (EKICRODISC, A Cro LC3CR, Nihon Pall Ltd., Tokyo, Japan) was used to filter the mixed solution. Subsequently, a 20-μL aliquot of the filtrate was injected into an HPLC system, consisting of two columns: HPLC-1 (MCI GEL CA08F, 7 μm, 1.5 × 120 mm; solvent A, 60 μL/min) and HPLC-2 (Insertsil ODS-3, 3 μm, 4.6 × 250 mm; GL Sciences Inc., Tokyo, Japan; solvent B, 0.7 mL/min). The HPLC-1 column was maintained at 50 °C, whereas the HPLC-2 column was set at 38 °C. A 20-μL aliquot of the urine sample was injected into the HPLC-1 column using a sampling injector. The 8-OHGuo fraction was automatically collected based on its retention time and injected into the HPLC-2 column, which was equipped with an electrochemical detector (ECD-300, Eicom Co., Kyoto, Japan; applied voltage: 550 mV). The solvents used included solvent A, consisting of 5% acetonitrile in 18.8 mM H_2_SO_4_, and solvent B, consisting of 9 mM K_2_HPO_4_, 25 mM KH_2_PO_4_, 0.7 mM EDTA•2Na, and 7% methanol. Urinary 8-OHGuo levels were expressed as ratios relative to the urinary creatinine content, measured using a UV detector at 235 nm.

### Statistical analysis

Statistical analysis was performed using the Student’s *t*-test to determine individual differences. Data were analyzed using the Statistical Package for Social Sciences, Statistics version 29.0 (IBM SPSS, IBM Corp., Armonk, NY, USA). Results are presented as mean ± SD, with statistical significance set at *p* < 0.05.

## Results

**Fig. 1:**
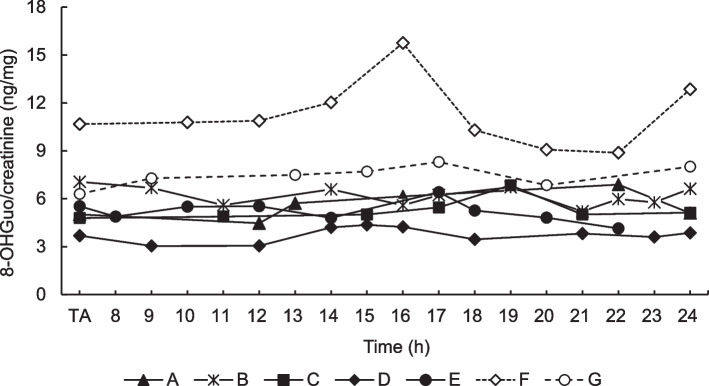
Diurnal variation in urinary 8-OHGuo levels. The levels were measured in five non-smokers (A–E), one smoker (F) and one ex-smoker (G) from the time of awakening to 24:00. Data presented represent typical examples for one day’s data of the participants (A–G)

The diurnal variations in urinary 8-OHGuo levels are presented in Fig. 1. No significant differences were observed in urinary 8-OHGuo levels at any time point between the non-smokers (Fig. 1A–E) and ex-smoker (Fig. 1G). By contrast, notable fluctuations were observed in the diurnal 8-OHGuo levels for the smoker (Fig. 1F). The coefficient of variation (CV) for the diurnal changes ranged from 9.6% to 15.7% for the five non-smokers (Fig. 1A–E), was 18.7% for the smokers (Fig. 1F), and 9.2% for the ex-smoker (Fig. 1G). The average 8-OHGuo level in the smoker was markedly higher (11.25 ng/mg creatinine) than that in non-smokers (3.37–6.19 ng/mg creatinine), whereas the average 8-OHGuo level in the ex-smoker (7.42 ng/mg creatinine) was intermediate between that of the smoker and non-smokers. Individual urinary 8-OHGuo levels remained relatively stable throughout the day, although a notable variation was observed in the smoker (Fig. 1). In summary, each individual maintained a characteristic value despite some diurnal fluctuations. These results suggest that the 8-OHGuo level in a spot urine sample can effectively reflect an individual’s characteristic value as a nonsmoker or ex-smoker for a given day. For smokers, the 8-OHGuo levels in morning spot urine samples may be more representative. However, the reason underlying the two peaks observed among smokers at 16:00 and 24:00 h remains unclear.

**Fig. 2:**
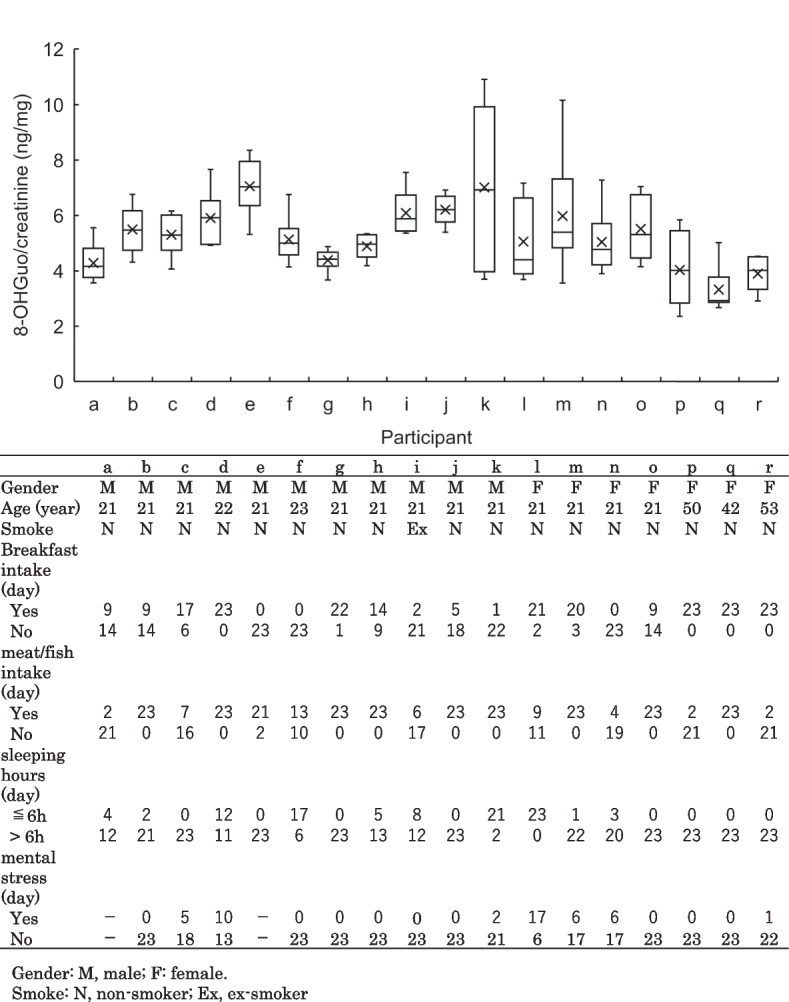
Ranges of urinary 8-OHGuo levels in spot urine samples collected from participants (*n* = 18) over 23 consecutive days. Urine samples were collected at the time of awakening (a–r). The center line in each box represents the median, while the whiskers show the minimum and maximum values, and the cross in each box represents the mean value

**Fig. 3:**
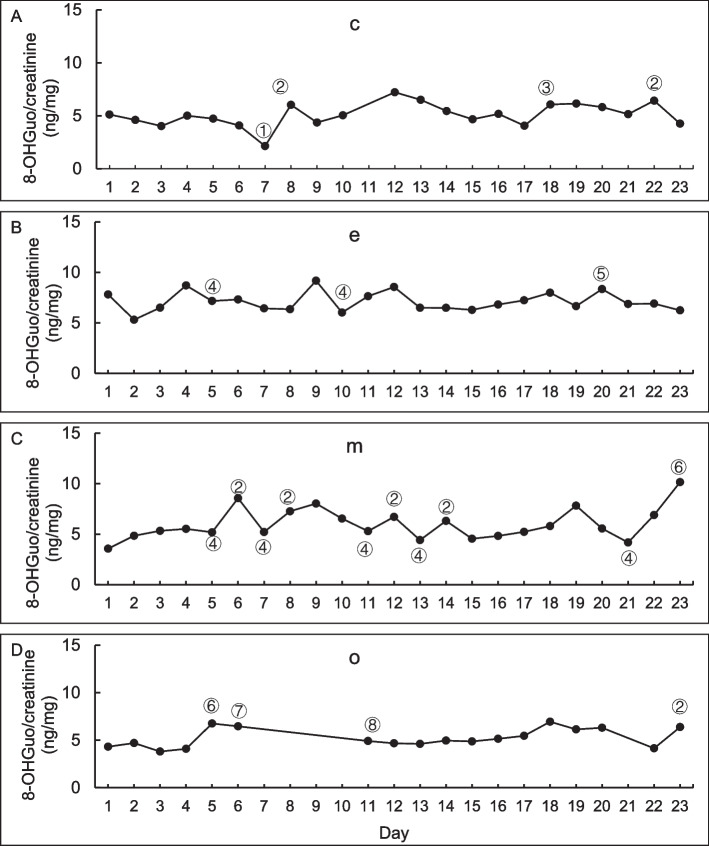
Typical daily variations in urinary 8-OHGuo in four participants over a period of 23 consecutive days. **A** participant c; **B** participant e; **C** participant m; **D** participant o (participant letter codes correspond to those in Fig. 2). Notations indicate: 1. relaxation; 2. sleep deprivation; 3. headache; 4. meat or fish intake; 5. smoked 16 cigarettes; 6. mental stress; 7. pre-menstrual period; 8. post-menstrual period. Urine samples were collected at the time of awakening

**Fig. 4:**
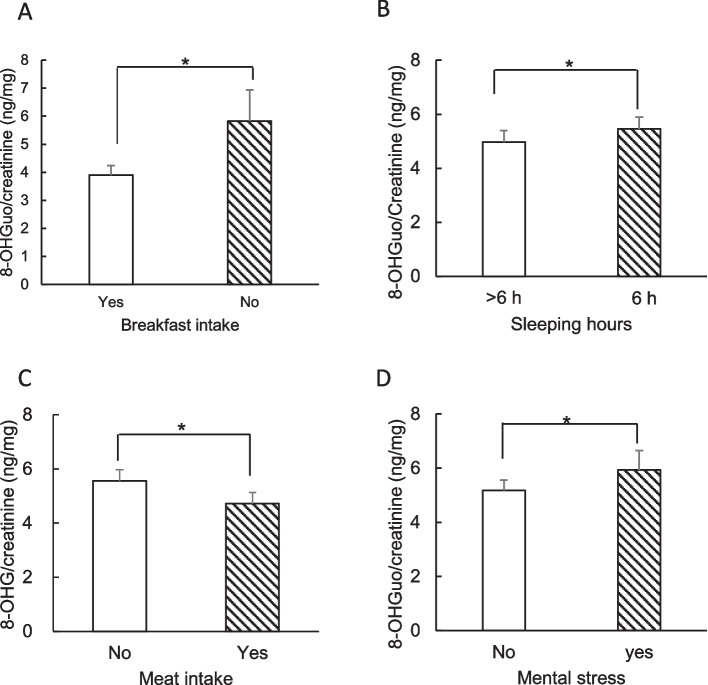
Relation between urinary 8-OHGuo levels and lifestyle factors. Urine samples were collected at the time of awakening. **A** Breakfast-eating habits (“Yes” group: *n* = 4; “No” group: *n* = 3, *p* = 0.029), *t*-test; **B** sleep duration (*n* = 7, *p* = 0.039), paired *t*-test; **C** Comparison of 8-OHGuo levels in participants the day before and after meat intake (*n* = 5, *p* = 0.024), paired *t*-test; **D** Mental stress (*n* = 5, *p* = 0.027), paired *t*-test. Each value represents mean ± standard deviation. * *p* < 0.05

The daily fluctuations in 8-OHGuo levels over 23 consecutive days for the 18 participants are displayed in Fig. 2. Each plot illustrates the individual average and median levels of urinary 8-OHGuo for 23 consecutive days, as well as the minimum and maximum values recorded. The CV of the daily variations for the 18 participants ranged from 9.2% to 40.5%. The range between the minimum and maximum values differed among individuals, with 8-OHGuo levels fluctuating within a specific range for each participant. In Fig. 3, the daily fluctuations in urinary 8-OHGuo levels for four participants (c, e, m, and o) over 23 days are illustrated, with participants selected because of their relatively complete lifestyle records. The individual 8-OHGuo levels fluctuated within a certain range. Based on lifestyle records, Fig. 3 depicts several events recorded on the day prior to urine collection, including 1. relaxation; 2. sleep deprivation; 3. headache; 4. meat or fish intake (for participants primarily vegetarian in daily life); 5. smoked 16 cigarettes (for participants who usually smoked 1–7 cigarettes per day); 6. mental stress; 7. pre-menstrual period; and 8. post-menstrual period. Participants who skipped breakfast daily exhibited significantly higher urinary 8-OHGuo levels than those who consumed breakfast daily (*p* = 0.029; Fig. 4A). Among participants who accurately reported their sleep duration, the average urinary 8-OHGuo levels were significantly higher when their sleep duration was less than 6 h (*n* = 7, *p* = 0.039; Fig. 4B). Furthermore, urinary 8-OHGuo levels were lower in participants when they consumed meat on the day prior to testing than when they did not consume any meat (*n* = 5, *p* = 0.024; Fig. 4C). Additionally, urinary 8-OHGuo levels were higher in five participants when they experienced mental stress (*n* = 5, *p* = 0.027; Fig. 4D).

## Discussion

In the current study, no significant differences were found in individual diurnal urinary 8-OHGuo levels among spot urine samples collected from the time of awakening until midnight (24:00) in non-smokers and the ex-smoker, suggesting that each participant had a distinct characteristic level of 8-OHGuo. Measurements of urinary 8-OHGuo levels in spot urine samples collected every 4 h have demonstrated that these levels are not significantly affected by circadian rhythms throughout the day [25, 27]. Therefore, the timing of sample collection does not appear to be critical for urinary 8-OHGuo levels and does not significantly impact the assessment of nucleic acid oxidation in urinary samples. Mao et al. [28] reported that 8-OHGuo levels, corrected for creatinine in random urine samples, were consistent with the levels in 24-h samples for both healthy participants and patients with renal disease. The 8-OHGuo levels in random and 24-h samples across age groups did not significantly differ, suggesting that random samples can effectively represent systemic oxidative stress for the entire day.　In the current study, the smoker exhibited higher levels and greater intraday fluctuations in urinary 8-OHGuo levels compared with those exhibited by non-smokers and the ex-smoker. Zhao et al. [29] reported that smokers showed significantly higher urinary 8-OHGuo levels than non-smokers. The presence of significant diurnal variations presents a valuable opportunity for health maintenance by encouraging lifestyle changes, such as quitting smoking, and assessing the risk of oxidative stress–related diseases.

A previous study indicated that urinary 8-OHGuo levels adjusted for urinary creatinine show relatively high day-to-day variations, with a CV ranging from 10.4% to 14.3% in urine samples collected once daily over 5 consecutive days [30]. Diurnal and day-to-day variations do not seem to significantly impact large-population epidemiological studies. However, from an individual perspective, such variation may have a more significant impact when using these measures as clinical indicators for evaluating individual oxidative stress. In the current study, consistent with previous reports [7, 29, 31], the results indicated that several lifestyle factors—such as smoking, mental stress, sleep deprivation, physical condition, inadequate nutrition, and menstrual cycles in women—are probably causally related to day-to-day variations in 8-OHGuo levels. Notably, we found that skipping breakfast led to an increase in urinary 8-OHGuo levels (Fig. 4A), indicating that this unhealthy dietary habit induces oxidative stress and could increase the risk of developing lifestyle-related diseases or cancers. Skipping breakfast is known to increase the risk of cardiovascular disease and mortality [32]. Therefore, oxidative stress induced by skipping breakfast may potentially contribute to an increased risk of cardiovascular disease and mortality. Regarding the effect of sleep duration, urinary 8-OHGuo levels increased with sleep deprivation (< 6 h) (Figs. 3 and 4B). Villafuerte et al. [33] found that sleep deprivation reduced catalase and superoxide dismutase activities in the spleen, brain, and liver. Similar variations in urinary 8-OHdG levels have been observed earlier [34]. An unbalanced diet and reduced intake of meat or fish increases urinary 8-OHdG levels, whereas the consumption meat or fish decreases these levels [34–36]. In the current study, urinary 8-OHGuo levels decreased with meat or fish intake (Fig. 4C), which is similar to a finding by Yahia et al. [37], who reported that the intake of fish protein improved tissue antioxidant status in rats. Kawai et al. [19] reported that certain fish products (e.g., Iriko, Shirasu, and Maroboshi) and ham contain 4.5–406 ng/g of 8-OHGuo. Furthermore, when mice were administered a 500 ng/mL 8-OHGuo solution, the excretion-to-intake ratio of 8-OHGuo was found to be 9%–15% [38]. These findings suggest that the consumption of fish and meat may not directly result in 8-OHGuo excretion through urine but instead improve the body’s antioxidant status and reduce oxidative stress levels. A moderate intake of meat or fish does not cause oxidative damage to DNA or RNA. In the current study, mental stress was associated with increased urinary 8-OHGuo levels, while relaxation appeared to decrease these levels (Fig. 3), which is similar to a finding by Jorgensen et al. [39] who reported that urinary 8-OHGuo levels increased with increasing severity of depression, suggesting a correlation between severe depression and increased oxidative RNA damage.

## Conclusions

In summary, the results of this study suggest that despite no significant diurnal changes in urinary 8-OHGuo being observed, an individual’s baseline oxidative stress status can be assessed by measuring spot urine samples over several days. Additionally, daily values of urinary 8-OHGuo may reflect the impact of lifestyle factors on oxidative stress, thereby providing valuable insights for preventing lifestyle-related diseases and assessing risks associated with environmental and occupational exposures. However, the limited number of participants in this study prevented statistical validation of the experimental results. Future research with large sample sizes is necessary to confirm these findings.

## Data Availability

The data supporting the manusctipt are included within the manuscript or available upon request.

## Abbreviations

8-OHGuo: 8-Hydroxyguanosine
8-OHdG: 8-Hydroxy-2′-deoxyguanosine
HPLC-ECD: High-performance liquid chromatography with electrochemical detection
ROS: Reactive oxygen species
